# The Northern Indiana and Southern Michigan Society

**Published:** 1894-11

**Authors:** 


### THE NORTHERN INDIANA AND SOUTHERN MICHIGAN SOCIETY.

The ninth semi-annual meeting of the Northern Indiana and Southern Michigan Homœopathic Medical Association was held in Elkhart, Ind., September 27th, Dr. W. D. Chaffee, Vice-President, in the chair. Members present: Drs. R. N. Morris and S. T. Mitchell, Constantine; C. H. Myers, W. D. Chaffee, and R. L. Stine, South Bend; Geo. L. Shoemaker, Nappanee; W. A. Whippy, Goshen; M. H. Criswell, Edwardsburg; H. C. Allen, T. C. Duncan, and W. A. Smith, Chicago; T. C. Buskirk, White Pigeon; A. L. Fisher, Porter Turner, H. A. Mumaw, and Mrs. S. M. Devor, Elkhart. Visiting physicians: Drs. C. L. Dreese, Goshen, and T. V. Roy, Valparaiso.

The meeting was called to order at eleven o'clock by the Second Vice-President, Dr. Morris, in the absence of Dr. Chaffee, and after roll-call the minutes of the previous meeting were read by the Secretary, Dr. H. A. Mumaw, and approved. The names of Drs. T. S. Hoyne, J. F. Beaumont, and E. W. Sawyer, Chicago; U. W. Reed, North Manchester; Ada B. Morgan, South Bend; J. E. Barbour, Bristol, and Alpha R. Leib, Elkhart, were presented for membership. The Chairman appointed Drs. Turner, Duncan, and Criswell a committee on credentials. The report was favorable and the election unanimous.

Reports of the necrologist and delegates from other societies were in order. In the absence of the necrologist, Dr. Thomas, this report was deferred until the next meeting. Dr. Hoyne spoke in his apt style of the good work being done by the Chicago Society of Materia Medica; Dr. Beaumont had an encouraging word for the new Chicago Homœopathic Medical Society; Dr. Duncan, the untiring worker in the interests of the American Institute of Homœopathy, spoke of its flourishing condition; Dr. Smith added a few encouraging words for the Chicago Baptist Hospital.

Reports of Bureaus were next in order. Chairmen: Surgery, Dr. Myers; Ophthalmology, Dr. Kreider; Practice, Dr. Duncan; Materia Medica, Dr. Buchtel; Gynæcology, Dr. Grove. The following papers were read and fully discussed by all the members present: "Artificial Diseases," by Dr. Sawyer; "Chronic Rhus Poisoning," by Dr. Allen; "Surgical Preliminaries," by Dr. Howard Crutcher, Chicago (read by the Secretary); "Nervous Diseases Due to Eye-Strains," by Dr. Beaumont; "Beauties of Homœopathy," by Dr. Whippy; "New Cure for

Consumption," by Dr. Duncan; "Local Applications as Curative Measures," by Dr. Smith; "A Therapeutical Ollapodrida," by Dr. Fisher. Dr. Hoyne, the veteran editor and lecturer, as well as practitioner, acted as general critic. Chairmen of Bureaus for the next meeting were appointed by the President, as follows: Surgery, Dr. Fisher; Ophthalmology, Dr. Beaumont; Materia Medica, Dr. Buskirk; Practice, Dr. Criswell; Gynæcology, Dr. Morgan.

A bill of $16.60 for printing, postage, and other expenses was presented by the Secretary; allowed and ordered paid.

Dr. T. V. Roy, a native of India, was elected an honorary member. He spoke of the sanitary condition of his country in an interesting and emphatic manner.

The meeting was one of great interest and profit. Adjourned. —*Daily Truth, of Elkhart, Ind., Sept. 28th*, 1894.

